# Supplementary material for: Potential Chemotherapeutic Effect of Selenium for Improved Canceration of Esophageal Cancer
Source: Int J Mol Sci. 2022 May 14;23(10):5509. doi: 10.3390/ijms23105509 (PMC9145868; doi:10.3390/ijms23105509)
Supplement: Supplementary file 1 [file ijms-23-05509-s001.zip › ijms-1717762-supplementary.pdf]

Method S1:

Cell Counting Kit-8 (CCK8) Assay and Morphological Examination

Briefly, ESCC cell lines were seeded between 500 and 3000 cells/well, depending on their growth rate. For KYSE 150 (500 and 1500 cells/well), KYSE 520 (1000 and 3000 cells/well) and CSEC 216 (1000 and 3000 cells/well) were seeded onto 96-well plates, always with the first column of wells without cells (blank), and allowed to grow overnight. The next day, the medium was aspirated. Cells were exposed to 200 µL/well of freshly prepared medium containing Sodium selenite, MSA, MSC, and Se-Met for 2 days and 6 days, and freshly Se compounds were added, to the new culture medium, to the cells on days 1, 3, and 5. After that, 10 µL of CCK solution was added to each well and was incubated at 37˚C for 1–2 h. Then, the absorbance was read using a Microtiter-plate reader (Bio-Rad, California, USA) at a wavelength of 450 nm. Normal cells were used as the control group, and the cell viability of the control group was assumed to be 100 %. For each treatment, the data were normalized to the absorbance value of untreated cells. Each experiment was conducted in triplicate and repeated at least three times. Representative morphological responses to selenium exposure were documented with a Polaroid camera at x10 magnification under a phase-contrast inverted microscope (Zeiss, Jena, Germany).

Quantification of Intracellular ROS

This method is based on the oxidation of DCFH-DA by ROS resulting in the formation of the fluorescent compound 2, 7-dichloroflorescin (DCF). DCFH-DA is a non-polar compound that readily diffuses into the cells. Intracellular esterases hydrolyze it to the non-fluorescent derivative 2, 7-dichloroflorescin, which is polar and trapped within the cells. In the presence of ROS, this compound is oxidized to DCF. Briefly, ESCC cell lines were seeded between 1000 and 3000 cells/well, depending on their growth rate. For KYSE 150 (1×10^3 and 3×10^3 cells/well), KYSE 520 (2×10^3 and 4×10^3 cells/well) and CSEC 216 (2×10^3 and 4×10^3 cells/well) were seeded onto 96-well plates overnight. Then next day, cells were exposed to different doses of Se compounds (Selenite 6 µM, MSA 2 µM, MSC 100 µM, and Se-Met 100 µM) for 1 and 3 days. After Se treatment, DCFH-DA was added into the culture medium to a final concentration of 10 µM, and the cells were incubated for another 30 min. at 37˚C. The dye was aspirated, and the cells were washed twice with 100 µL of PBS. Fluorescence intensity was measured using a microplate reader (EX/EM = 485/535 nm (Molecular devices filter max F5 filter microplate reader). % ROS was expressed as relative fluorescence (RFU) of the positive control according to Equation (1). Blank absorbance was measured in wells containing DCFDA

without cells. Fluorescence images were visualized with an inverted fluorescence microscope (Zeiss, Germany).

$$RFU = (F - Fb)/ (Fctr+ - Fb) \times 100$$

Immunofluorescence Staining

Briefly, cells were harvested when they reached 80% confluence. Then KYSE 150 (2×10^4 and 1×10^4 cells/well), KYSE 520 (3×10^4 and 15×10^3 cells/well), CSEC 216 (3×10^4 and 15×10^3 cells/well), human esophageal immortalized epithelial cell lines NE2 (1×10^5), and NE6 (1×10^5) were inoculated onto a coverslip in a 24-well plate and incubated for 24 h at 37˚C. Following incubation, ESSC cell lines were treated with different concentrations of Se compounds (Selenite 6 µM, MSA 2 µM, MSC 100 µM, and Se-Met 100 µM) for 1 and 3 days at 37˚C and human esophageal immortalized epithelial cell lines were preincubated with various concentrations of (Selenite 6 µM, MSA 2 µM, MSC 100 µM, and Se-Met 100 µM) for 2 h. Cells were stimulated with 800 µM H_2O_2 for 8 h for NF-κB and 16 h for phospho-H2AX and 8-OHdG in the presence of Se compounds. Subsequently, cells were fixed with 4% paraformaldehyde at room temperature for 30 minutes, permeabilized with 0.5% Triton X-100 at room temperature for 30 minutes, and washed with PBS three times after each treatment. Primary antibodies of phospho-H2AX (1:300 dilution), 8-OHdG (1:500 dilution), and NF-κB (1:100 dilution) were applied to the slide and incubated in a humidified box at 4˚C overnight. A secondary goat anti-rabbit antibody conjugated with Alexa Fluor® 488 was applied after PBS washing three times for 5 min. each and put in a black wet box with shading and then incubated in a humidified chamber at 37 ˚C for 30 min. After incubation, the slide was washed with PBS and mounted using antifade mounting with DAPI. Cells were then visualized under a fluorescence microscope (Zeiss, Jena, Germany) utilizing a 488 nm excitation. Fluorescence images were captured by ZEN imaging software (ZEN 2011; Carl Zeiss). Analysis of NF-κB subcellular localization by determining the integrated fluorescence intensity of the NF-κB stain that overlapped with the DAPI stain, which corresponds to the nuclear contribution of the signal, and subtracting this from the total integrated fluorescence intensity to determine the cytoplasmic fraction. The nuclear fluorescence intensity of 8-OHdG and γ-H2AX foci number from each cell was quantified using the Cell Profiler and ImageJ software (National Institutes of Health, Bethesda, MD, USA). The nuclear/cytoplasmic fluorescence intensity of NF-κB and the nuclear fluorescence intensities of 8-OHdG and γ-H2AX foci number were measured in at least 200 cells per treatment group. The mean fluorescence intensity was used as the level of DNA damage.

Immunohistochemical Staining (IHC)

Briefly, the FFPE sections were de-paraffinized with xylene followed by rehydration in descending grades of ethanol. Heat-mediated antigen retrieval was performed using a vegetable steamer containing 10 mM citrate buffer (pH 6.0) at 125˚C for 3 min followed by cooling at room temperature and washing with phosphate-buffered saline (PBS). Endogenous peroxidase activities were blocked with 3% hydrogen peroxide for 15 minutes followed by 10% normal goat serum for 30 min. at 37˚C. To eliminate the non-specific staining. Next, FFPE sections were incubated with antibodies specific for Ki-67 (1:500 dilution), γ-H2AX (1:100 dilution), 8-OHdG (1:300 dilution), CD45 (1:300 dilution), CD4 (1:400 dilution) and CD8 (1:400 dilution) at 4˚C overnight. After washing with PBS, FFPE sections were incubated with secondary antibody at 37˚C for 30 min. DAB (Diaminobenzidine) was applied for 5 minutes for visualization. FFPE sections were counterstained with Harris hematoxylin, dehydrated through ethanol, cleared with xylene, and mounted with cover glasses. Immunohistochemical staining was observed blindly by two independent pathologists. Images were captured using a Leica IM50 microscope at ×400 magnification, and five different fields for each index were selected in each sample. The expression of Ki-67 and γ-H2AX was quantified by the number of positive nuclei and total nuclei in images counted. The corresponding immunostaining positive rates were computed as positive nuclei/total nuclei ×100%. Integrated optical density (IOD) was tested by Image-Pro Plus 6.0 software due to the nuclear and cytoplasmic immunoreactivity of 8-OHdG. The inflammatory markers (CD45, CD4, and CD8) are membrane expressions; we count the positive cell number in each field by Image-Pro Plus 6.0 software.

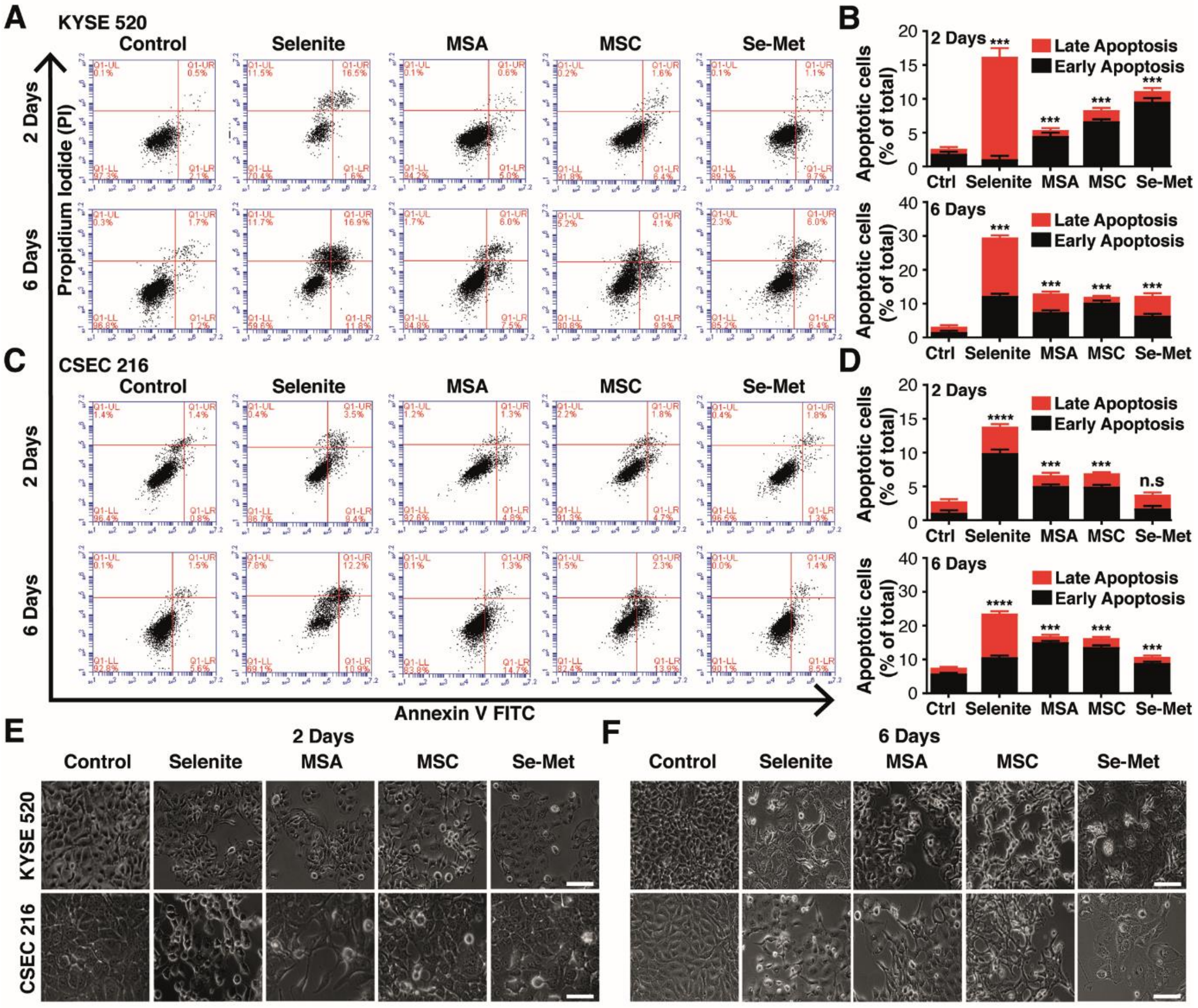

Figure S1. Selenium induces apoptosis in ESCC cell lines. Cells were treated with Selenite (6 μM), MSA (2 μM), MSC (100 μM), and Se-Met (100 μM) for 2 and 6 days. Cell apoptosis was measured by Flow cytometry. **(A)** Flow cytometry dot plot figures of apoptotic cells in KYSE 520. **(B)** apoptotic cells represent the percentage of Annexin V single positive and Annexin V/PI double-positive cells after 2 and 6 days in KYSE 520. **(C)** Flow cytometry dot plot figures of apoptotic cells in CSEC216. **(D)** apoptotic cells represent the percentage of Annexin V single positive and Annexin V/PI double-positive cells after 2 and 6 days in CSEC 216. In each dot plot figure, the upper left quadrant resembles necrotic cells; the upper right quadrant contains the later apoptotic cells, which are positive for Annexin V and propidium iodide (PI); the lower left quadrant shows viable cells, which exclude PI and Annexin V; the lower right quadrant denotes the early apoptotic cells, Annexin V positive and PI negative. Phase-contrast photomicrograph depicted representative morphological responses of KYSE 520 and CSEC 216 cell lines at **(E)** 2 days and **(F)** 6 days. Differences are significant at (***p < 0.001, ****p < 0.0001 vs. untreated control, n.s, non-statistically significant, $n \geq 3$). Data are

expressed as mean ± SD from three ($n = 3$) independent experiments Difference with $p \leq 0.05$ was considered statistically significant. Scale bar, 20 μm.

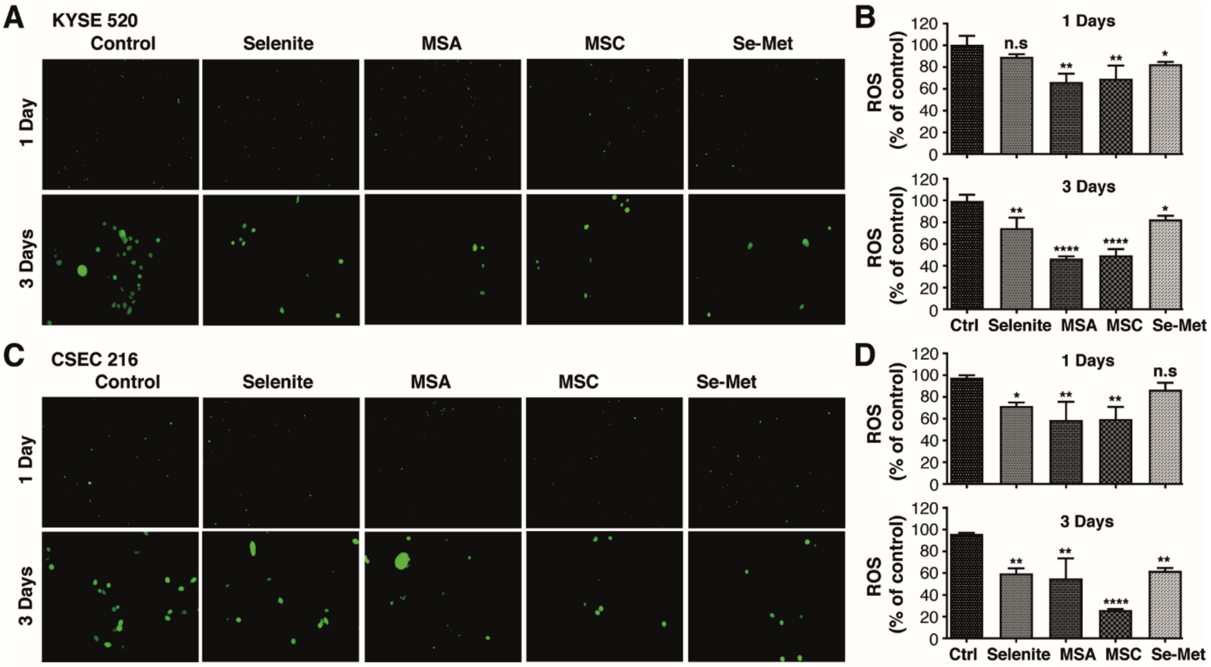

Figure S2. Selenium diminishes intracellular ROS levels in ESCC cell lines. Cells were treated with Selenite (6 μM), MSA (2 μM), MSC (100 μM), and Se-Met (100 μM) for 1 and 3 days. **(A)** The level of intracellular ROS in KYSE 520 cells was stained by DCFH-DA and observed under Zeiss Vert A1 fluorescence microscope. **(B)** Statistical results were read by fluorescent plate reader in KYSE 520 cells. **(C)** The level of intracellular ROS in CSEC 216 cells was stained by DCFH-DA and observed under Zeiss Vert A1 fluorescence microscope. **(D)** Statistical results were read by fluorescent plate reader in CSEC 216 cells. Differences are significant at (*$p < 0.05$, **$p < 0.01$, ****$p < 0.0001$ vs. untreated control, n.s, non-statistically significant, $n \geq 3$). Data are expressed as mean ± SD from three ($n = 3$) independent experiments. The difference with $p \leq 0.05$ was considered statistically significant.

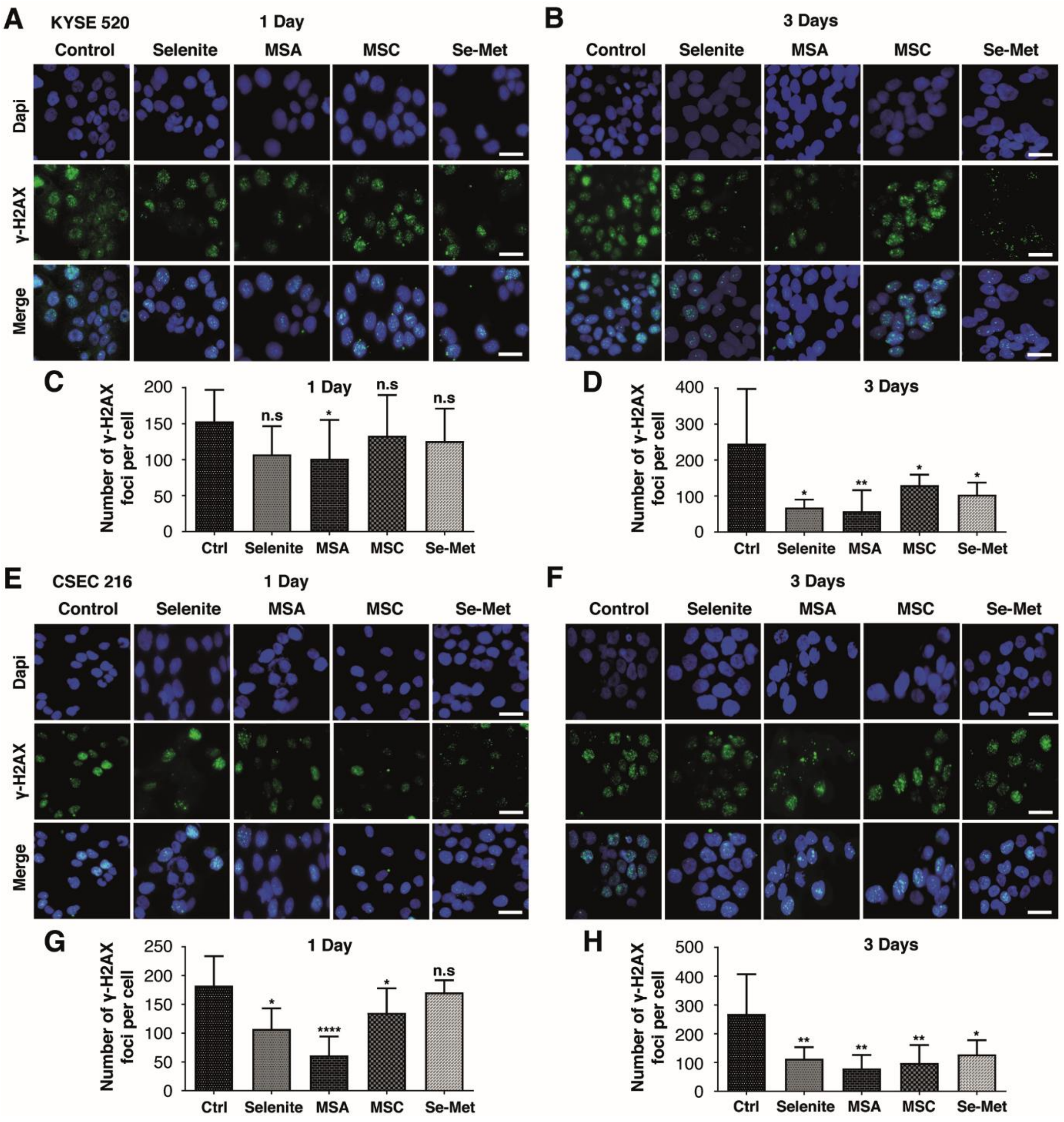

Figure S3. Selenium prevents γ-H2AX foci formation in ESCC cell lines. Cells (KYSE 520 and CSEC 216) were treated with Selenite (6 μM), MSA (2 μM), MSC (100 μM), and Se-Met (100 μM) for 1 and 3 days. γ-H2AX expression was visualized by immunofluorescence using primary specific antibodies and Alexa Fluor 488-conjugated secondary antibodies. Nuclei were stained with DAPI. **(A, B)** Representative images of γ H2AX immunostaining after 1 day and 3 days in KYSE 520. **(C, D)** Columns represent the number of γ-H2AX foci/cell for each treatment after 1 day and 3 days in KYSE 520. **(E, F)** Representative images of γ H2AX immunostaining after 1 day and 3 days in CSEC 216. **(G, H)** Columns represent the number of γ-H2AX foci/cell for each treatment after 1 day and 3 days in CSEC 216. The number of γ-H2AX foci/cell was determined by counting at least 100 cells on 10 fields randomly selected

for each sample. Differences are significant at (*p < 0.05, **p < 0.01, ****p < 0.0001 vs. untreated control, n.s, non-statistically significant, $n \geq 3$). Data are expressed as mean ± SD from three (n = 3) independent experiments Difference with $p \leq 0.05$ was considered statistically significant. (Magnification, ×40; scale bar=20 μm).

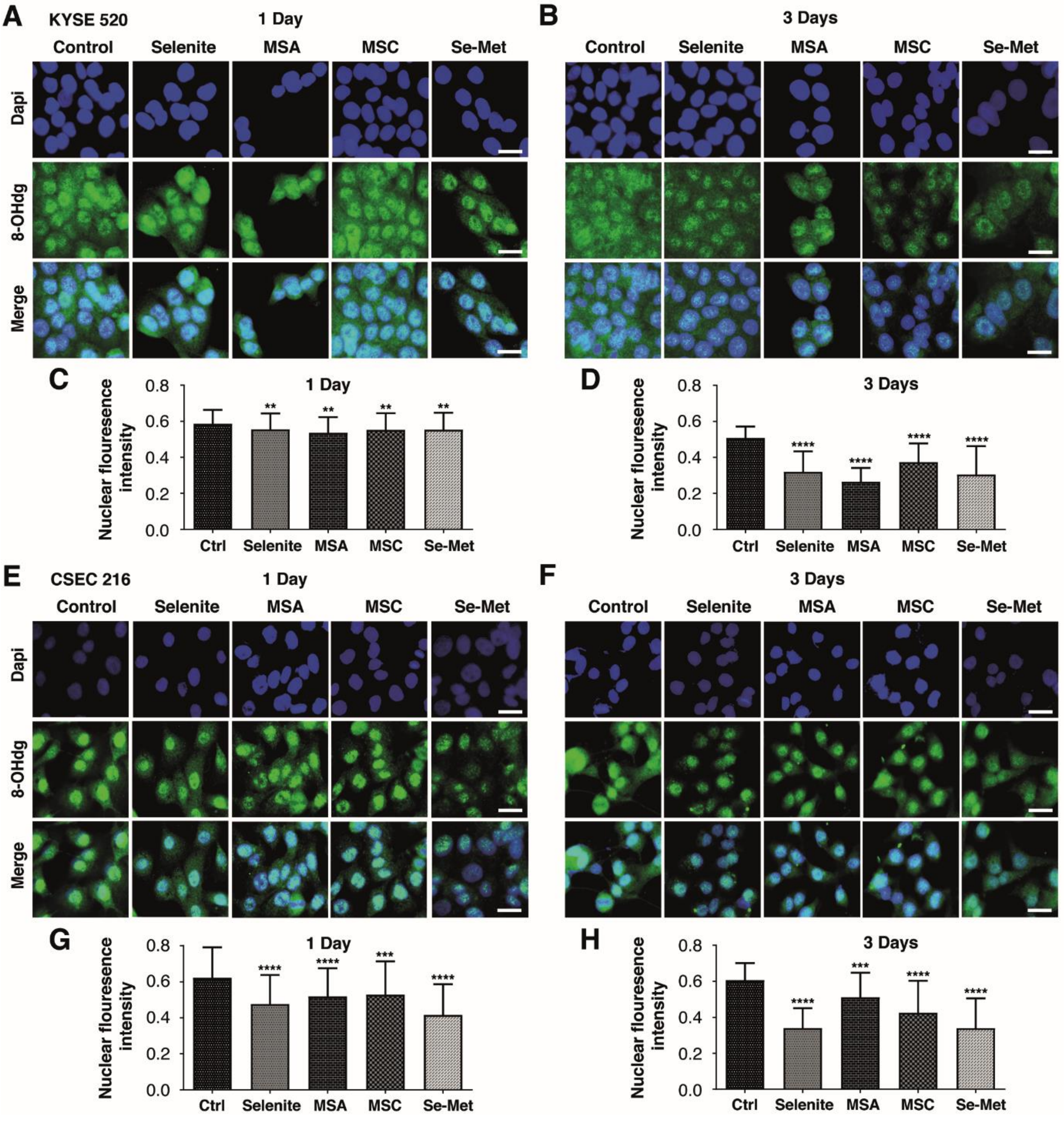

Figure S4. Selenium reduces the 8-OHdg intensity level in ESCC cell lines. Cells (KYSE 520 and CSEC 216) were treated with Selenite (6 μM), MSA (2 μM), MSC (100 μM), and Se-Met (100 μM) for 1 and 3 days. 8-OHdg expression was visualized by immunofluorescence using primary specific antibodies and Alexa Fluor 488-conjugated secondary antibodies. Nuclei were stained with DAPI. **(A, B)** Representative images of 8-OHdg immunostaining after 1 day and

3 days in KYSE 520. **(C, D)**. Histograms show nuclear fluorescence intensity signal quantification in the nuclear of each treatment after 1 day and 3 days in KYSE 520. **(E, F)** Representative images of 8-OHdg immunostaining after 1 day and 3 days in CSEC 216. **(G, H)** Histograms show nuclear fluorescence intensity signal quantification in the nuclear of each treatment after 1 day and 3 days in CSEC 216. The nuclear fluorescence intensity/cell was determined by counting at least 100 cells on 10 fields randomly selected for each sample Differences are significant at (**p < 0.01, ***p < 0.001, ****p < 0.0001 vs. untreated control, n.s, non-statistically significant, $n \geq 3$). Data are expressed as mean ± SD from three ($n = 3$) independent experiments. The difference with $p \leq 0.05$ was considered statistically significant. (Magnification, ×40; scale bar=20 μm).

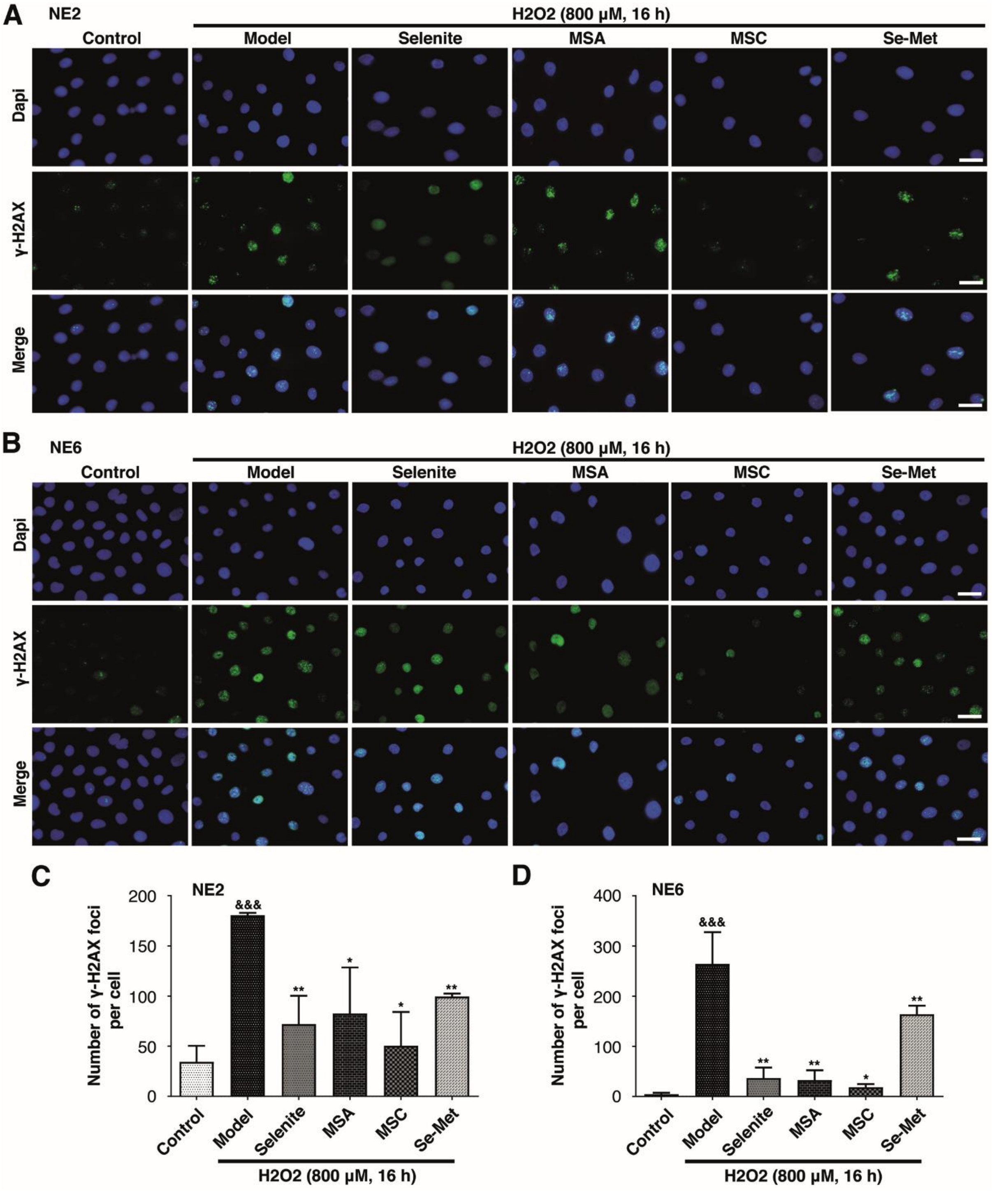

Figure S5. Selenium prevents γ-H2AX foci formation in esophageal immortalized epithelial cell lines. Cells (NE2 and NE6) were pre-treated with Selenium compounds, Selenite (6 μM), MSA (2 μM), MSC (100 μM), and Se-Met (100 μM) for 2 h and then co-cultured with (800 μM) H_2O_2 for 16 h. The DNA double-strand breaks were assessed by fluorescence-based immunocytochemistry using specific antibodies for γ-H2AX. **(A, B)** Representative images of γ-H2AX fluorescence in NE2 and NE6 cell lines. **(C, D)** Columns represent the number of γ-

H2AX foci/cell for each NE2 and NE6 cell line treatment. The number of γ-H2AX foci/cell was determined by counting at least 100 cells on 10 fields randomly selected for each sample. Differences are significant at ($^{\&\&\&}$p < 0.001 vs untreated group, *p < 0.05, **p < 0.01, vs. H_2O_2 group, $n \geq 3$). Data are expressed as mean ± SD from three (n = 3) independent experiments Difference with p ≤ 0.05 was considered statistically significant. (Magnification, ×40; scale bar=20 μm).

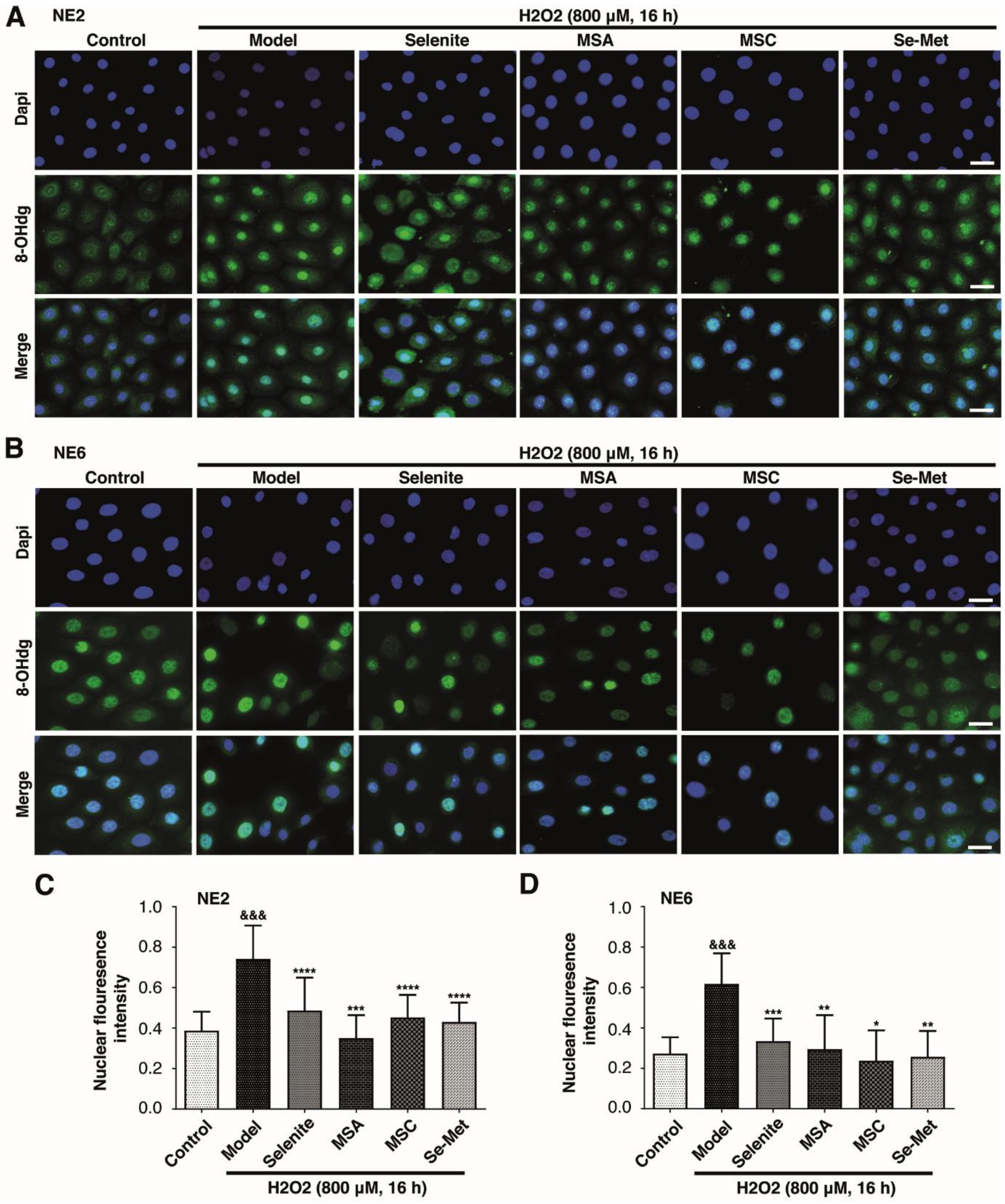

Figure S6. Selenium reduces 8-OHdg intensity level in esophageal immortalized epithelial cell lines. Cells (NE2 and NE6) were pre-treated with Selenium compounds, Selenite (6 µM), MSA (2 µM), MSC (100 µM), and Se-Met (100 µM) for 2 h and then co-cultured with (800 µM) H_2O_2 for 16 h. 8-OHdg expression was visualized by immunofluorescence using primary specific antibodies and Alexa Fluor 488-conjugated secondary antibodies. Nuclei were stained with DAPI. The nuclear fluorescence intensity/cell was determined by counting at least 100 cells on 10 fields randomly selected for each sample. **(A, B)** Representative images of 8-OHdg fluorescence in NE2 and NE6 cell lines, respectively. **(C, D)** Columns represent the nuclear fluorescence intensity/cell level for each treatment in NE2 and NE6 cell lines, respectively. Differences are significant at ($^{\&\&\&}p < 0.001$ vs untreated group, $^{*}p < 0.05$, $^{**}p < 0.01$, $^{***}p < 0.001$, $^{****}p < 0.0001$ vs. H_2O_2 group, $n \geq 3$). Data are expressed as mean ± SD from three (n = 3) independent experiments Difference with $p \leq 0.05$ was considered statistically significant. (Magnification, ×40; scale bar=20 µm).
